# Rumen In Vitro Fermentation and In Situ Degradation Kinetics of Winter Forage Brassicas Crops

**DOI:** 10.3390/ani9110904

**Published:** 2019-11-01

**Authors:** José Daza, Daniel Benavides, Rubén Pulido, Oscar Balocchi, Annick Bertrand, Juan Keim

**Affiliations:** 1 Animal Production Institute, Faculty of Agricultural Sciences, Universidad Austral de Chile, Valdivia PO Box 567, Chile; jose_daza11@hotmail.com (J.D.); obalocch@uach.cl (O.B.); 2 Graduate School, Faculty of Agricultural Sciences, Universidad Austral de Chile, Valdivia PO Box 567, Chile; dabepez89@hotmail.com; 3 Animal Science Institute, Faculty of Veterinary Sciences, Universidad Austral de Chile, Valdivia PO Box 567, Chile; rpulido@uach.cl; 4 Soils and Crops Research and Development Centre, Agriculture and Agri-Food Canada, Québec City, QC G1V 2J3, Canada; Annick.Bertrand@AGR.GC.CA

**Keywords:** kale, swede, volatile fatty acids, degradation rates

## Abstract

**Simple Summary:**

Winter brassica crops such as kales and swedes are used to supply feed in times of seasonal shortage. However, to the best of our knowledge, there is little information about the fermentation characteristics of these forages in the rumen. This study assessed the nutrient concentration, in vitro fermentation and in situ rumen degradation characteristics of *Brassica oleracea* (L.) ssp. *acephala* (kales) and *Brassica napus* (L.) ssp. *napobrassica* (swedes). The kales and swedes both showed different nutrient concentrations and fermented fast and extensively in the rumen. However, in vitro fermentation of swedes resulted in lower acetate and greater proportions of butyrate and propionate. Varieties of swedes showed more differences in terms of degradation and fermentation in the rumen compared to kale varieties.

**Abstract:**

The aim of the present study was to evaluate the nutritional value, the rumen in vitro fermentation, and the in situ degradation of *Brassica oleracea* (L.) ssp. *acephala* (kales) and *Brassica napus* (L.) ssp. *napobrassica* (swedes) for winter use. Five varieties of each brassica were used in three field replicates and were randomized in a complete block nested design. All forage varieties were harvested at 210 days post-sowing to analyze the chemical composition, in vitro gas production, volatile fatty acid (VFA) production and in situ dry matter (DM) and crude protein (CP) degradability. Kales presented higher DM and neutral detergent fiber (NDF) content (*p* < 0.01), whereas swedes showed higher CP, metabolizable energy (ME), glucose, fructose, total sugars, NFC, and nonstructural carbohydrate (NSC) content (*p* < 0.01). The kale and swede varieties differed in their CP and sugar concentrations, whereas the kale varieties differed in their DM and raffinose content. The rates of gas production were higher for swedes than for kales (*p* < 0.01). No differences between the brassica species (*p* > 0.05) were observed in the total VFA production, whereas kales had a higher proportion of acetate and swedes had higher proportions of butyrate (*p* < 0.05). Only the swede varieties showed differences in VFA production (*p* < 0.05). The soluble fraction “a”, potential and effective in situ DM degradability were higher in swedes (*p* < 0.01), but kales presented greater DM and CP degradation rates. Differences were observed between brassica species in the chemical composition, degradation kinetics, and ruminal fermentation products, whereas differences among varieties within species were less frequent but need to be considered.

## 1. Introduction

Brassicas such as kales (*Brassica oleracea* (L.) ssp. *acephala*) and swedes (*Brassica napus* (L.) ssp. *napobrassica*) are used for ruminant feed during winter [1], which is a season with low pasture growth in humid temperate regions [2]. These forages can offer high dry matter (DM) production and nutritional quality in a short time, which is related to high metabolizable energy (ME), water-soluble carbohydrates (WSC), and low neutral detergent fiber (NDF) content [3,4]. Winter brassicas have been used successfully in sheep [4], dry cows [5], and lactating dairy cows [6]. In addition, forage brassicas have an environmental advantage; they reduce the amount of enteric methane (CH_4_) per unit of DM intake compared to ryegrass pasture [4,7]. Although nutrient concentrations in brassicas have been widely described, the nutritive value of these forages depends on the quantity of nutrients available to the animal, which is determined by fermentation processes [8] and the presence of secondary compounds such as glucosinolates and S-methyl-cysteine sulfoxide that are present in brassicas [9], thus meaning animal responses can be affected.

Complementary evaluation methods, such as the ruminal digestibility of nutrients or products of ruminal fermentation and metabolism, have been suggested to determine the real nutritive value of forages [10,11]. The ruminal in situ incubation technique is considered a reference method to estimate degradation parameters, when adjusted to suitable nonlinear models [12]. These parameters are used by feeding evaluation models to estimate nutritive value, nutrient supply, and animal performance [8]. On the other hand, the in vitro gas production technique (IVGPT) allows the determination of fermentation kinetics [13]; estimates of DM, protein, and fiber degradation; ruminal volatile fatty acid (VFA) content; and microbial protein synthesis [14]. The popularity of in vitro gas production (GP) stems mainly from the ability to exercise experimental control, the capacity to nondestructively screen a large number of substrates, the kinetic information obtained, and relatively low costs [10]. Thus, IVGPT offers a unique tool for researchers to address a wide range of nutritional issues in ruminants [15].

Whereas degradation kinetics and ruminal fermentation of summer brassica species (rape and turnip) and varieties [16] have been reported in the literature, few reports exist on the effect of winter brassica species (kales and swedes) and varieties on the in situ degradation kinetics and fermentation end products. For example, Sun et al. [4] have observed that sheep fed swedes showed modified VFA profiles in their rumen fluid and lowered methane yield in contrast with those fed kales or perennial ryegrass. Keogh et al. [1] have reported no effects on the rumen VFA concentration from increases in the dietary proportion of kales in the diets of dry cows. Valderrama and Anrique [17] have reported DM and crude protein (CP) degradation kinetics of kale leaves; however, to the best of our knowledge, such data have not been reported for swedes. Moreover, the nutritive value of brassicas varies among species and varieties within species [3,16], and, therefore, information is still lacking about rumen fermentation and the kinetics of winter brassica species such as kales and swedes.

Hence, the aim of this study was to determine the nutritive value of forage brassica species (kales and swedes) and varieties for winter use, based on their nutrient concentration, in vitro ruminal fermentation, and in situ rumen degradation kinetics.

## 2. Materials and Methods

All animal procedures were performed in accordance with the UK Animals (Scientific Procedures) Act and associated guidelines, and approved by the Animal Ethics Committee of the Austral University of Chile (approval number 144/2013).

### 2.1. Site and Experimental Design

This experiment was carried out at the Agricultural Research Station (39°47′ S, 73°13′ W) of the Austral University of Chile on a Typic Hapludand soil with an initial water pH of 5.8, Olsen-P of 19.1 mg/kg, exchangeable potassium of 214 mg/kg, and aluminum saturation of 3.1% (measured for the first 20 cm of the soil profile).

Prior to soil preparation and sowing, the weeds were controlled chemically with glyphosate at a dosage of 2025 g/ha of active ingredient. Two brassica species were evaluated (kales and swede), and five varieties were sown for each species: Caledonian (K1), Elba (K2), Sovereign (K3), Regal (K4), and Coleor (K5) for the kales and Major Plus (S1), Aparima Gold (S2), Highlander (S3), Dominion (S4), and Invitation (S5) for swedes. The plot sizes were 6 m by 4 m, with three replicates for each variety, and plots were arranged in field blocks. The varieties were established in October 2014 at a seed dosage of 4.0 (kales) and 1.5 kg/ha (swedes). Fertilizers were applied at sowing to correct any soil nutrient deficiencies. A fertilizer mixture (7 % N-30 % P_2_O_5_ -12 % K_2_O) at doses of 500 kg/ha (35 kg N/ha, 150 kg P_2_O_5_/ha, and 60 kg K_2_O/ha) and 46 kg/ha boronatrocalcite were applied at sowing. After emergence, weeds were controlled chemically by applying Lontrel 3A (clopyralid 475 g of active ingredient (a.i.)/L) and Tordon 24 k (picloram 240 g a.i./L) at doses of 300 and 200 cc/ha respectively. Five weeks after sowing, when the plants had two or three leaves, 125 kg N/ha (urea) was applied, and in January 2015, applications of 750 cc/ha of Aramo (tepraloxydim 200 g a.i./L) and 200 cc/ha of karate (50 g a.i./L Lambda-cyhalothrin) were made.

During the trial, five cuts were made, with an approximate interval of 30 days between cuts, with the first harvest occurring at 90 days after plant emergence. In each cut, 4 m^2^ of each crop was harvested. The kale varieties were cut to 20 cm above the ground level, the swede varieties were collected manually, and soil attached to the roots was removed. Plants were weighed and then separated into the main components (leaf and stem for kales and leaf and bulb for swedes). The samples were then dried in a forced-air oven at 60 °C for 48 h for determination of the dry matter (DM).

Samples for nutrient concentrations and in vitro and in situ incubations were harvested at 210 days post-sowing, and the plants were separated into their morphological components (leaf and stems for kale varieties and leaf and bulbs for swede varieties) before being chopped and then being frozen at −20 °C. Later, they were lyophilized (Virtis 10-45 MR-BA, Gardiner, New York, NY, USA) and then ground (Wiley mill, 158 Arthur H. Thomas, Philadelphia, Pennsylvania, PA, USA) to 5 mm for in situ incubations and to 1 mm for nutrient concentrations analyses and in vitro gas production. For the in vitro and in situ incubations, samples of brassica species were composed of a leaf to stem ratio of 35:65 for kales and a leaf to bulb ratio 30:70 for swedes. The ratios were the average proportions of organ components at harvest obtained in this study.

### 2.2. In Situ Incubations

Three dry Holstein-Friesian cows (one for each block) fitted with ruminal cannulas (4′ Pliable Rumen Cannula w/Stopper and U Bolt, Ankom Technologies, Macedon, New York, NY, USA) were used. At the time of the experiment, the ruminal pH (6.55 ± 0.32) of each cow was measured. Cows were offered grass silage (7.5 kg DM), summer turnips (4.5 kg DM) and commercial concentrate (2.0 kg DM). Samples of each variety were incubated in duplicate (~4 g DM) in Dacron bags (10 cm by 20 cm; pore size of 40–60 µm) and sealed. Up to 20 bags were deposited inside a lingerie bag (30 cm by 40 cm in size). Brassica samples from each block were incubated in a different cow and a control sample (commercial concentrate) was incubated in each cow to evaluate cow-to-cow variation.

Prior to incubation in the rumen, the bags were soaked in warm water (40 °C) for 20 min. Nine incubation times were considered: 0, 2, 4, 8, 10, 12, 14, 24, and 48 h. The samples corresponding to 0 h were not introduced into the rumen and were used to determine the soluble fraction. After the incubation, the bags containing the residue were removed from the rumen and were washed under running cold water until no further color appeared; then, they were frozen at −20 °C for 24 h to stop fermentative activity. Thereafter, the bags were defrosted, thawed in water at 4 °C, and washed with a commercial washing machine for 30 min at a “normal” wash setting. Finally, residues were oven-dried at 60 °C for 48 h. The residues were weighed, the DM was calculated by placing the samples in an oven at 105 °C for 12 h, and the CP concentration was determined to calculate nutrient loss.

A correction for small particle loss was made as follows. The samples of each brassica variety (1.5 g) were weighed in a beaker. Then, 40 mL of tap water was added and the mix was stored at room temperature (20 °C) for 1 h; afterward, the mix was filtered through a nitrogen-free filter paper and washed eight times with 20 mL of water. The residues were oven-dried and analyzed individually. Degradation parameters (a, b, and c), potential degradation (PD) and effective degradability (ED) were corrected according to Hvelplund and Weisbjerg [18].

### 2.3. In Vitro Incubations

Duplicates (1 g) of each sample were incubated in 160 mL glass bottles. Each brassica variety, a control (commercial concentrate) sample and two blanks (bottles without substrate) were used. Subsequently, 85 mL of Goering-Van Soest medium and 4 mL of reducing agent (NaOH 2.5 mM and cysteine-HCl 2.5 mM) were added at 39 °C under continuous gasification (CO_2_) to maintain anaerobic conditions and the bottles were covered with rubber stoppers and aluminum seal.

The inoculum was extracted from two dry Holstein-Friesian cows with ruminal cannulas with a live weight (PV) of 560 ± 20 kg and a ruminal pH of 6.6 ± 0.53; at the time of the extraction, the animals were offered the same diet as in the in situ trial. Rumen fluid was obtained before the cows were fed in the morning and was stored in a thermos flask to preserve the temperature until being transferred to the laboratory. Once there, the fluid was filtered through four layers of cheesecloth while being maintained at a temperature of 39 °C and under a constant flow of CO_2_. Rumen fluids from the two donor cows were mixed in equal proportions and then inoculated (10 mL) into the bottles. After inoculation, the bottles were placed in a water bath at 39 °C under continuous horizontal movement at 50 rpm.

Once the rumen fluid was inoculated, the initial gas was extracted from the bottles. The gas pressure in the headspace of the bottles above atmospheric pressure was measured manually with a pressure transducer (PCE Instruments, Tobarra, Albacete, Spain) at 2, 3, 4, 5, 6, 8, 10, 12, 18, 24, 36, and 48 h, and the volume of gas produced was measured by extraction using syringes connected through a three-way Luer valve from the bottles until the visual display of the transducer read zero, and once the volume of gas produced was recorded, it was eliminated. Fermentations were stopped after 48 h by placing the bottles on ice. Each field block (five varieties of kales and five varieties of swedes) was incubated at different runs. Thus, the first block was incubated at a first run, those corresponding to block number two were incubated at the second run, and samples from block three were incubated at the third run. A control standard (commercial concentrate) was incubated at each incubation run to control the day-to-day variation.

Once the in vitro incubation was finished, the samples were kept on ice to stop fermentative processes and residue duplicates from each sample were collected and then centrifuged at 15,000× *g* and 4 °C. After centrifugation, 0.9 mL of the supernatant was extracted to determine the VFA concentrations with a GG-2010 gas chromatograph (Shimadzu Corporation, Kyoto, Japan).

### 2.4. Analyses

The dry matter content was measured by weighing the samples before and after drying with a forced-air oven, initially at 60 °C for 48 h and then at 105 °C for 12 h. The CP concentration was determined by combustion (Leco Model FP-428, Nitrogen Determinator, Leco Corporation, St Joseph, Minnesota, MI, USA) based on the DUMAS method (nitrogen × 6.25); digestible organic matter on a dry matter basis (DOMD) was measured according to Tilley and Terry [19]; neutral detergent fiber (aNDF) was measured by using a heat stable amylase [20]; and ash and ether extract (EE) were analyzed according [21] (Methods ID 942.05 and ID 920.39 for ash and EE respectively). Sugars (raffinose, sucrose, glucose, and fructose) were analyzed by Waters ACQUITY ultrahigh-performance liquid chromatography (UPLC, Waters, Milford, Massachusetts, MA, USA), and starch quantification was determined by colorimetric detection of non-soluble residues after enzymatic digestion with amyloglucosidase according to Pelletier et al. [22]. The sum of sugars and starch yielded the content of total nonstructural carbohydrates (NSC). An estimation of the combined organic acids plus neutral detergent soluble fiber (OA + NDSF) was calculated according to Hall et al. [23], where OA + NDSF = NFC − NSC. Non-fibrous carbohydrates were calculated as follows: NFC = 100 − CP − aNDF − EE − ash + neutral detergent insoluble crude protein (NDICP).

### 2.5. Calculations

The in situ disappearance of DM and CP was determined using the non-linear model described by Ørskov and McDonald [12] to determine the potential degradation according to the exponential model

PD = *a* + *b* × (1 − *e*^−*kt*^)

where *a* is the soluble fraction (fraction washed out at *t* = 0; this value resulted from the incubation of 0 h bags corrected for particle loss and fixed into the model), *b* is the insoluble but potentially degradable fraction, *k* is the degradation rate (per hour), and *t* is the time (h).

The effective degradability (ED) was calculated assuming a fractional passage rate (kp) of 2%, 5%, and 8% per hour according to the following equation:

ED = *a* + *b* × *c*/(*c* + kp)

These parameters were corrected for the losses of small particles which are degraded in a similar way to the particles remaining in the bag, as reported by Hvelplund and Weisbjerg [18].

After correcting for gas production of the blanks, the obtained GP data were adjusted to the generalized Michaelis-Menten model without a lag phase [11], as seen in the equation

GP = A × [*T^n^*/(*T^n^* + *K^n^*)]

where GP is the gas production at time *T*, A is the asymptote of GP (mL), *n* is the determined value of the shape of the curve, and *K* is the time taken to produce half of A.

The following parameters were calculated according to Groot et al. [24] and France et al. [11], i.e.,

fermentation rate at half-life (C) = *n*/(2 × *K*)
maximal fermentation rate (MDR) = (*n* − 1)^((*n*−1)/*n*)/k^
time to ferment *x*% of the substrate (*t_x_*) = *K* × ((X/(1 − X))^(1/n)^)

where X = 0.25, 0.75, and 0.90 of A.

### 2.6. Statistical Analyses

Parameters of in vitro GP, in situ degradation kinetics, VFA content and nutrient concentration were averaged for analytical replicates and analyzed with the MIXED procedure of SAS (SAS Institute, Cary, North Carolina, NC, USA).

Data were analyzed under a nested design, with three replicates organized in complete randomized blocks. Varieties were nested within species and the random effect of the field replicate was included as a block. When significant differences (*p* < 0.05) were found, the Tukey-Kramer multiple-comparison test was used in the LSMEANS procedure statement in SAS.

## 3. Results

### 3.1. Nutrient Concentration and Sugar Profile

Kales had greater concentrations of DM, EE (+4 g/kg), and aNDF (+123 g/kg) than swedes (*p* < 0.01; Table 1). Swedes showed greater CP (+25 g/kg; *p* < 0.01) and total sugar concentrations (+75 g/kg; *p* < 0.01) than kales, as well as individual sugars, such as glucose and fructose, NSC, OA + NDSF and DOMD. Raffinose and sucrose concentrations were greater in kales (*p* < 0.01). The ash and starch concentrations did not vary between the species (*p* > 0.05).

The kale varieties differed in their DM, CP, EE, raffinose, glucose, fructose, sugars, starch, and NSC concentrations (*p* < 0.01). Coleor had greater concentrations of CP (+30 g/kg) than Regal; Elba and Sovereign greater EE than Coleor; Coleor greater raffinose than Coledonian and Sovereign; and Regal and Coledonian greater concentrations of glucose and fructose than Sovereign. Finally, Regal showed greater concentrations of total sugars than Elba and Sovereign; Sovereign and Coleor greater concentrations of starch than Coledonian; and Regal the greatest NSC concentration of all varieties (231 g/kg), whereas Elba and Sovereign had the lowest (190 g/kg and 194 g/kg, respectively).

Furthermore, the swede varieties differed in their CP, glucose, fructose, sugars, starch, and NSC concentrations (*p* < 0.01). The concentrations of ash, aNDF, sucrose, OA + NDSF, and DOMD for varieties of both species were not different (*p* > 0.05). Invitation showed greater concentrations of CP (+30 g/kg) than Aparima Gold. Major Plus and Highlander showed the greater concentration of glucose, fructose, and sugars (280 g/kg and 288 g/kg, respectively, compared to 233 g/kg, 241 g/kg, and 234 g/kg in Aparima Gold, Dominion and Invitation). For starch, Aparima Gold and Dominion had greater concentrations (32 g/kg and 26 g/kg, respectively) than Highlander (7 g/kg). Finally, the concentrations of NSC in Major Plus and Highlander (291 g/kg and 295 g/kg, respectively) were greater than that for Invitation.

### 3.2. In Situ Degradation Parameters

Dry matter degradation parameters differed between brassica species (Table 2) except for the insoluble but potentially degradable fraction “b” (*p* > 0.05). Swedes had a higher soluble fraction “a” compared to kales (591 g/kg and 499 g/kg, respectively; *p* < 0.01) but a lower degradation rate “c” (0.25 h^−1^ and 0.34 h^−1^, respectively; *p* < 0.01). However, swedes showed greater PD (+90 g/kg) and ED with ruminal passage rates of 2%, 5%, and 8% per hour compared to kales (*p* < 0.01). No significant differences were found in the in situ degradation parameters for the kale varieties (*p* > 0.05). Within the swede varieties, fraction “a” was greater for Major Plus (634 g/kg) compared to Aparima Gold (545 g/kg), Dominion (581 g/kg), and Invitation (586 g/kg; *p* < 0.01).

For the in situ CP degradation parameters, a species effect was observed on the fractional degradation rate “c”, with kales having a faster degradation rate compared to swedes (0.48 h^−1^ and 0.36 h^−1^, respectively; *p* < 0.01), whereas no brassica species effect was observed on the other in situ CP degradation parameters with average values of 559 g/kg, 380 g/kg, and 939 g/kg for “a”, “b”, and PD, respectively (*p* > 0.05). Fractions “a” and “b” were affected by the kale varieties: Coleor presented a higher “a” than Regal and Caledonian (+159 g/kg and +139 g/kg, respectively; *p* < 0.01). On the other hand, Caledonian and Regal had a greater “b” fraction compared to Coleor (*p* < 0.05). Effective degradability at 8% per hour of Sovereign (904 g/kg) and Coleor (900 g/kg) were higher than that of Regal (839 g/kg; *p* < 0.05). No effects for the swede varieties were found for any of the CP degradation parameters (*p* > 0.05).

### 3.3. In Vitro Fermentation Products

The in vitro GP parameters were affected by the brassica species, whereas for varieties within species no effect was observed (Table 3). The GPs at 24 h and 48 h and “A” were higher for swedes compared to kales (256 mL/g, 285 mL/g, and 285 mL/g against 233 mL/g, 260 mL/g, and 261 mL/g DM, respectively; *p* < 0.01). Fermentation rate parameters “C” and “MDR” (h^−1^) were slightly faster for swedes (0.15 h^−1^) compared to kales (0.14 h^−1^; *p* < 0.05). However, no differences were observed for the time to fermentation of 25%, 50%, and 75% of the substrate (h), whereas 90% of the substrate was fermented 1.1 h earlier for swedes (*p* < 0.05).

For the total volatile fatty acids (tVFA) concentrations, the propionate and branched-chain VFA (BCVFA) molar proportions of tVFAs showed no effects for brassica species (*p* > 0.05; Table 4). The acetate molar proportions of tVFAs (+48 mmol/mol) and A:P ratio were greater for kales, whereas the fermentation of swedes increased the molar proportions of butyrate (+33 mmol/mol). Kale varieties showed no differences for tVFA and the relative proportions of the different VFAs, whereas the tVFAs and the relative proportion of acetate and butyrate were affected by swede varieties. Major Plus showed a higher concentration of tVFAs (61.3 mM) compared to Dominion (45.5 mM), and Major Plus, Aparima Gold, and Invitation had higher relative proportions of acetate (555 mmol/mol, 564 mmol/mol, and 572 mmol/mol, respectively) compared to Dominion, which showed a higher relative proportion of butyrate (162 mmol/mol) than Aparima Gold and Invitation (128 mmol/mol and 120 mmol/mol, respectively).

## 4. Discussion

### 4.1. Nutrient Concentration

The current study reports the nutritive value of five varieties of two winter forage brassica species that are used to complement the feeding base used in grazing systems when pasture availability is not sufficient to fulfill livestock requirements. Forage brassicas have a higher demonstrated DOMD than perennial pastures; Sun et al. [4], for example, have reported that the DOMD of kales and swedes (883 g/kg and 918 g/kg DM) is 25% higher than that in perennial ryegrass pastures (703 g/kg DM). However, DOMD values observed by the current study are lower than those previously reported for kales and swedes (727 g/kg and 831 g/kg DM).

Sun et al. [4] and Westwood and Mulcock [3] have observed that kales contain higher values of DM, EE, and aNDF than swedes, which is similar to the effect observed in the present study. Regarding CP, swedes showed a higher concentration (136 g/kg) compared to kales (111 g/kg). Westwood and Mulcock [3] have reported similar values for swedes (137 g/kg) but lower values for kales (97 g/kg); however, they have observed that CP is highly variable among kale varieties, ranging from 63 g/kg to 138 g/kg, which is in accordance with our study where the CP concentration of the kale varieties ranged from 88 g/kg to 128 g/kg. These differences are mainly associated with the leaf to stem ratio, as forage brassicas concentrate more CP in their leaves [25]. For example, Valderrama and Anrique [17] have reported CP concentrations of 225 g/kg in kale leaves.

The content of sugars was similar to that reported by Sun et al. [4], with swedes having a higher concentration of sugars compared to kales. Winter brassicas have higher sugars compared to grass-based permanent pastures and perennial ryegrass, where values range from 73 g/kg to 118 g/kg when harvested during winter and early spring, the times when winter brassicas are used [26,27].

The amount of raffinose and sucrose were higher in kales; however, these amounts were not enough to compensate for the higher concentration of fructose and glucose in swedes. Importantly, varieties of both kales and swedes differed in the concentrations of fructose and glucose. In the case of kales, the Regal concentrated almost twofold the amounts of glucose and fructose compared to Sovereign, whereas for swedes, the Major Plus and Highlander concentrated approximately 20 g/kg and 30 g/kg more than the other three varieties. This approach is important, as it considers the type and amount of sugars demonstrated to affect fermentation in the rumen and the end products such as the VFAs [28].

Additionally, the concentration of OA + NDSF was evaluated, and this was higher for swedes (327 g/kg DM) than for kales (291 g/kg DM). This parameter was evaluated in summer brassicas by Keim et al. [16], who found it to be 313 g/kg and 314 g/kg DM for turnip and rape, respectively. Neutral detergent soluble fiber is mainly composed of galactans, β-glucans, soluble hemicelluloses, and pectic substances [23], while organic acids (OA) are mainly carbohydrate derivatives and may contain lactate, citric acid cycle components and secondary plant compounds such as oxalate and shikimate [23]. Previous studies have reported concentrations of pectins between 77 g/kg and 129 g/kg DM for brassicas in general [29]. Likewise, Sun et al. [4] have reported 80 g/kg and 69 g/kg DM for kales and swedes, respectively. In addition to sugars, these components of the NDSF could have an effect on ruminal fermentation and VFA concentrations, and although NDSFs are readily and extensively broken down in the rumen, they do not mimic the pH-lowering effect of starch because they generally produce little or no lactate, and their fermentation ceases at low pH [30]. On the other hand, organic acids do not support microbial growth [23] and therefore have little impact on rumen metabolism.

### 4.2. In Situ Degradation Parameters

Brassica forages are characterized by their high concentration of readily fermentable carbohydrates and high digestibility [9], which is in accordance with the high soluble fraction and fast degradation rates observed in our study for both species. To the best of our knowledge, limited data exist from reports on degradation kinetics of kales [17] and no published literature has been found regarding the degradation kinetics of swedes. The greater soluble fraction observed in swedes is in accordance with its greater concentration of sugars and nonstructural carbohydrates compared to kales. Both, kales and swedes showed a fast degradation rate and extensive degradation of DM and CP compared with other forages used for livestock feeding during winter such as grass pastures, silages, and hay [31,32], and they showed a similar potential degradability but faster degradation rate compared to other winter fodder crops, such as fodder beets [33], and summer brassicas, such as turnips and forage rape [16]. The faster degradation rates observed in our study compared to those observed by Keim et al. [16] in summer brassicas and the values reported by Valderrama and Anrique [17] for kales might be explained by the composition of the diet offered to the cannulated cows. In this experiment, cows were offered brassicas in their diet, and, therefore, rumen microbes might have been adapted to degrade these kind of ingredients, which would have increased the fermentation rate [34].

Even though kales showed a faster degradation rate of crude protein than swedes, due to the similar soluble (a) and insoluble but potentially degradable (b) fractions, PD and ED were similar among species. The crude protein ED of kales was similar to the values reported by Valderrama and Anrique [17] when calculated at a passage rate of 2% per hour and was slightly greater with passage rates of 5% and 8% per hour because of the fast degradation rate observed in our study (0.48 h^−1^). Importantly, the CP fractions “a” and “b” differed among the kale varieties and need to be considered for the ration formulation models. This variation in degradation characteristics associated with genetic differences has been reported previously by Sun et al. [35] with perennial ryegrass. The varieties with greater CP concentration (Coleor) also showed the highest soluble fraction of crude protein.

### 4.3. In Vitro Fermentation

Higher 24 h, 48 h, and asymptotic GP in swedes reflects greater digestibility compared to kales, since total GP is an indicator of forage digestibility [36] and has been related to DM degradability [37], which is in accordance with the greater DM potential and effective degradability observed for swedes compared with kales. This difference may be attributed to the chemical composition, as forages with lower aNDF content and higher NSC, such as swedes, present greater GP [38]. As observed for DM potential and effective in situ degradability, the in vitro GP parameters were not affected by varieties within species, as a good correlation between the GP measurements and in situ degradability has been found [39]. Contrary to what was observed with in situ DM degradation rates (faster for kales than swedes), in vitro gas production rates (C and MDR) were faster for swedes. This is because in vitro gas production rates take into account the gas produced from the soluble fraction, whereas the in situ degradation rate comes only from the insoluble but potentially degradable fraction. Nevertheless, C and MDR values of both swedes and kales (0.14 and 0.15, respectively) demonstrated faster fermentation compared to other typical feedstuffs used for ruminant feeding during winter, such as concentrate (0.11 h^−1^ for both c and MDR), hay (0.03 and 0.05 h^−1^ for c and MDR), silage (0.07 and 0.08 h^−1^ for c and MDR) and pasture (0.09 and 0.10 h^−1^ for c and MDR) [40].

Fast fermentation of both kales and swedes is reflected by parameters t25, k, t75, and t90, where, for example, 90% of both species ferment in less than 23 h, mainly due to the presence of readily fermentable carbohydrates such as sugars and NDSF. Hall et al. [41] have shown that fermentation of NDSF is faster than fermentation from NDF, indicating that forages high in NDSF tend to exhibit a rapid fermentation.

In contrast to the GP, the total VFA production was similar among species. This finding could be because the digested OM is fermented to VFA and GP or converted to microbial protein and therefore total VFA and GP are not always well correlated [42]. Total VFA production was different across swede varieties and related to the in situ DM soluble fraction; that is, the Major Plus variety presented the greater soluble fraction and tVFA production, whereas Dominion showed the lowest values for both tVFA and DM soluble fraction. The acetate and acetate to propionate ratio were greater for kale, which is related to the high aNDF and lower NSC concentrations in kales compared to swedes, as has been reported previously [27,42]. In addition, the highest proportion of butyrate found in swedes coincides with the higher sugar concentration, since the fermentation of sugars generally increases the amount of butyrate in the rumen [28]. The greater acetate and lower butyrate proportions of tVFA for kales compared with swedes are in agreement with in vivo studies with sheep, where acetate was reduced from 515 mmol/mol to 412 mmol/mol, while butyrate was increased from 118 mmol/mol to 176 mmol/mol for sheep fed swedes compared with those fed kales [4].

Kale varieties had no effect on VFA production and the relative proportions of VFAs, whereas differences in the acetate and butyrate relative proportions of tVFA were observed for varieties of swedes. The varieties with greater butyrate relative proportions of tVFA were those with greater sucrose and fructose concentrations as well as a higher DM soluble fraction.

### 4.4. Implications

Although nutrient concentrations of winter brassicas and variations among varieties have been widely described [3] and their use in sheep [4], dry cows [5] and lactating dairy cows [6] have been reported, to the best of our knowledge few studies have evaluated the rumen fermentation processes of winter brassicas.

The main products of rumen fermentation are VFAs, and among these, propionate is a substrate for gluconeogenesis and is the main source of glucose in the animal, whereas the non-glucogenic acetate and butyrate are sources for long-chain fatty acid synthesis [43]. Glucogenic and lipogenic nutrient supply and VFA profile have been associated with animal energy balance. It has been suggested that in lactating cows increased energy intake is channeled largely through increases in rumen production of butyric and propionic acids and their yield of ATP to the host animal [43,44]. From an environmental point of view, production of acetate and butyrate liberates hydrogen, whereas propionate serves as a net hydrogen sink [45]. Consequently, diets that increase propionate and decrease acetate in the rumen are often associated with a reduction in ruminal CH_4_ production [46].

Therefore, the results we obtained for in vitro fermentation end products may lead us to infer some animal responses. However, these extrapolations must be done carefully, because the mechanisms governing microbial efficiency and VFA molar proportions in vitro are not necessarily valid in vivo. For example, in the rumen itself, feed and microbial biomass are subject to passage and VFA subject to passage and absorption [10], processes that do not occur under in vitro conditions.

One of our main findings is that swedes increased the relative molar proportion of butyrate at the expense of acetate compared with kales, however there are differences among varieties of swedes that must be considered and depend basically on their sugar concentration and type of sugars. There is generally a positive association of feeding sugars, such as sucrose, and increased milk and milk fat production, which may be due to the greater molar proportion of butyrate produced from those sugars [44,47]. However, to the best of our knowledge no studies have reported milk production responses of dairy cows fed swedes. In vivo results reported by Sun et al. [4] are similar to our study, with total rumen VFA concentrations being similar for sheep fed swedes or kales and greater butyrate and lower relative molar proportions of acetate found for sheep fed swedes. Complementarily, dry matter intake (DMI) was greater when kales were offered but energy intake was greater when feeding swedes resulting in lower methane emissions; however, no animal responses were reported. Conversely, Keogh et al. [6] have observed no differences in body weight and body condition score change between pre-calving dairy cows supplemented with kales or swedes during the dry period, but rumen fermentation data was not shown in their work.

Finally, the in situ ruminal degradation parameters of kales and swedes generated in this study can be used by researchers and nutritionists in feeding evaluation models to estimate the nutritive value, nutrient supply, and animal performance of livestock fed with winter brassicas.

## 5. Conclusions

Relative to winter forage brassica crops, our study demonstrated high digestibility for ruminant feeding in times when pasture availability is low. Differences between the two species were observed in terms of chemical composition, gas production, and dry matter degradation parameters, with swedes exhibiting a faster and more extensive degradation due to their greater concentrations of readily fermentable carbohydrates and lower NDF, which resulted in a lower acetate to propionate ratio and greater butyrate concentrations in the rumen. Additionally, differences among varieties within species were observed and must be considered when selecting certain varieties for use. For example, varieties of swedes showed differences regarding their in situ DM soluble fraction and in vitro tVFA and acetate and butyrate relative proportions of tVFA, whereas kale varieties differed in their in situ soluble CP and insoluble but potentially degradable CP fractions. Continuing with studies under in vivo conditions when feeding winter brassicas, especially to lactating dairy cows, is important because less information is available.

## Figures and Tables

**Table 1:** Nutrient concentration (g/kg dry matter) of five varieties of kale (from K1 to K5) and swedes (from S1 to S5).

Parameter	Kales	Swedes	^1^ SEM	Kales					Swedes					^1^ SEM	*p* Value	
				K1	K2	K3	K4	K5	S1	S2	S3	S4	S5		Species	Variety
DM (g/kg sample)	122	74	0.3	108 ^b^	123 ^ab^	135 ^a^	107 ^b^	136 ^a^	71	81	72	71	77	0.6	<0.01	<0.01
Ash	79	77	0.2	88	83	76	73	74	74	75	73	81	82	0.5	NS	NS
CP	111	136	0.4	114 ^ab^	111 ^ab^	116 ^ab^	88 ^b^	128 ^ab^	127 ^ab^	123 ^b^	124 ^ab^	151 ^ab^	153 ^a^	0.9	<0.01	<0.01
EE	13	9	0.04	13 ^ab^	14 ^a^	14 ^a^	13 ^ab^	10 ^b^	10	9	9	9	9	0.09	<0.01	<0.05
aNDF	300	176	0.7	271	309	328	297	293	169	196	165	169	182	1.6	<0.01	NS
Raffinose	7	2	0.4	6 ^b^	7 ^ab^	5 ^b^	7 ^ab^	10 ^a^	2	1	2	2	2	0.9	<0.01	<0.01
Sucrose	59	15	4.6	47	52	75	50	70	11	17	12	16	22	10.4	<0.01	NS
Glucose	63	139	3.1	74 ^ab^	59 ^bc^	44 ^c^	83 ^a^	56 ^bc^	157 ^a^	127 ^b^	156 ^a^	123 ^b^	127 ^b^	6.8	<0.01	<0.01
Fructose	51	99	3.0	58 ^ab^	50 ^abc^	35 ^c^	69 ^a^	42 ^bc^	109 ^a^	88 ^b^	115 ^a^	100 ^ab^	82 ^b^	6.6	<0.01	<0.01
Sugars	180	255	5.3	185 ^ab^	168 ^b^	160 ^b^	209 ^a^	179 ^ab^	280 ^a^	233 ^b^	288 ^a^	241 ^b^	234 ^b^	11.9	<0.01	<0.01
Starch	25	19	3.7	14 ^b^	21 ^ab^	34 ^a^	22 ^ab^	31 ^a^	11 ^bc^	32 ^a^	7 ^c^	26 ^ab^	20 ^abc^	8.4	NS	<0.05
NSC	205	274	7.1	199 ^ab^	190 ^b^	194 ^b^	231 ^a^	210 ^ab^	291 ^a^	265 ^ab^	295 ^a^	267 ^ab^	253 ^b^	15.8	<0.01	<0.05
OA + NDSF	292	327	5.4	315	293	272	298	284	328	331	332	322	321	12.2	<0.01	NS
DOMD	727	831	0.7	740	715	698	732	749	841	823	841	828	824	1.7	<0.01	NS

^a, b, c^ within a row, different letters represent the significant differences at *p* value < 0.05. ^1^ SEM standard error of the mean; DM, dry matter; CP, crude protein; EE, ether extract; aNDF, neutral detergent fiber with a heat stable amylase; NSC, non-structural carbohydrates, representing the sum of sugars and starch; OA + NDSF, organic acids and neutral detergent soluble fiber; DOMD, digestible organic matter on DM basis. Kale varieties: K1, Caledonian; K2, Elba; K3, Sovereign; K4, Regal; K5, Coleor. Swede varieties: S1, Major Plus; S2, Aparima; S3, Highlander; S4, Dominion; S5, Invitation.

**Table 2:** In situ ruminal digestion kinetics, potential degradability (PD) and effective degradability (ED, considering a passage rate of 2%, 5%, and 8% per hour) of five varieties of kale (from K1 to K5) and swedes (from S1 to S5).

Parameter	Kales	Swedes	^1^ SEM	Kales					Swedes					^1^ SEM	*p* Value	
				K1	K2	K3	K4	K5	S1	S2	S3	S4	S5		Species	Variety
Dry matter degradation parameters																
a	499	591	0.6	519	499	479	504	491	634 ^a^	545 ^b^	612 ^ab^	581 ^b^	586 ^b^	1.3	<0.01	<0.01
b	370	368	0.6	363	374	374	376	363	349	387	352	367	384	1.4	NS	NS
*c*	0.34	0.25	0.02	0.35	0.32	0.37	0.30	0.35	0.22	0.29	0.28	0.22	0.22	0.05	<0.01	NS
PD	869	959	0.6	882	873	853	881	854	982	931	964	964	969	1.9	<0.01	NS
ED, 2%/h	846	931	0.8	860	849	831	855	835	952	907	940	919	936	1.7	<0.01	NS
ED, 5%/h	817	895	0.7	831	819	802	823	809	915	875	910	881	896	1.5	<0.01	NS
ED, 8%/h	793	867	0.7	807	794	779	797	786	886	848	885	851	864	1.5	<0.01	NS
Crude protein degradation parameters																
a	550	568	1.4	494 ^bc^	554 ^abc^	596 ^ab^	474 ^c^	633 ª	580	532	520	594	615	3.2	NS	<0.01
b	383	377	1.4	429 ^a^	387 ^ab^	354 ^ab^	431 ^a^	313 ^b^	384	398	390	361	353	3.1	NS	<0.05
*c*	0.48	0.36	0.02	0.53	0.42	0.53	0.43	0.47	0.34	0.40	0.41	0.31	0.32	0.05	<0.01	NS
PD	933	945	1.1	923	941	950	906	946	964	930	911	955	968	2.5	NS	NS
ED, 2%/h	918	927	1.0	909	924	937	888	933	943	912	898	935	947	2.3	NS	NS
ED, 5%/h	897	900	0.9	887	900	920	862	916	915	886	873	906	921	2.1	NS	NS
ED, 8%/h	878	877	0.9	867 ^ab^	879 ^ab^	904 ^a^	839 ^b^	900 ^a^	890	864	852	882	898	2.0	NS	<0.05

^a, b, c^ within a row, different letters represent the significant differences at *p* value < 0.05. ^1^ SEM standard error of the mean; a, soluble fraction; b, insoluble but potentially degradable fraction; c, fractional disappearance rate constant at which b is degraded. Kale varieties: K1, Caledonian; K2, Elba; K3, Sovereign; K4, Regal; K5, Coleor. Swede varieties: S1, Major Plus; S2, Aparima; S3, Highlander; S4, Dominion; S5, Invitation.

**Table 3:** In vitro gas production kinetics of five varieties of kales (from K1 to K5) and swede (from S1 to S5).

Parameter	Kales	Swedes	^1^ SEM	*p* Value	
				Species	Variety
24 h GP	232	256	3.0	<0.01	NS
48 h GP	260	285	3.1	<0.01	NS
A	261	285	3.3	<0.01	NS
K	6.1	6.1	0.08	NS	NS
C	0.14	0.15	0.01	<0.05	NS
MDR	0.14	0.15	0.01	<0.05	NS
t25	3.2	3.2	0.04	NS	NS
t75	11.8	11.5	0.2	NS	NS
t90	22.6	21.5	0.5	<0.05	NS

^1^ SEM standard error of the mean; 24 h GP (mL/g DM), gas production after 24 h of incubation; 48 h GP (mL/g DM), gas production after 48 h of incubation; A, asymptotic gas production (mL/g DM); K, time to ferment 50% of the substrate (h); C, degradation rate at half-life (per h); MDR, maximal degradation rate (per h); t25, t75, and t90, time to ferment 25%, 75%, and 90% of the substrate, respectively (h).

**Table 4:** In vitro rumen volatile fatty acids (VFA) of five varieties of kale (from K1 to K5) and swede (from S1 to S5).

Parameter	Kales	Swedes	^1^ SEM	Kales					Swedes					^1^ SEM	*p* Value	
				K1	K2	K3	K4	K5	S1	S2	S3	S4	S5		Species	Variety
*VFA concentration*																
Total VFA (mM)	52.5	51.2	1.7	52.6	49.6	49.1	56.9	54.5	61.3 ^a^	49.1 ^ab^	49.6 ^ab^	45.5 ^b^	50.4 ^ab^	3.8	NS	<0.05
Acetate (mmol/mol)	597	549	4.2	595	591	597	597	602	555 ^ab^	564 ^ab^	537 ^bc^	520 ^c^	572 ^a^	7.2	<0.05	<0.01
Propionate (mmol/mol)	245	258	1.9	245	249	243	243	244	255	254	266	258	257	4.3	NS	NS
Butyrate (mmol/mol)	106	139	2.3	107	104	106	111	103	141 ^ab^	128 ^b^	143 ^ab^	162 ^a^	120 ^b^	4.9	<0.01	<0.01
BCVFA (mmol/mol)	52	54	1.5	53	55	54	48	50	48	54	54	60	52	2.7	NS	NS
A:P ratio	2.4	2.1	0.04	2.4	2.4	2.5	2.5	2.5	2.2	2.2	2.0	2.0	2.2	0.09	<0.01	NS

^a, b, c^ within a row, different letters represent the significant differences at *p* value < 0.05. ^1^ SEM standard error of the mean; BCVFA, branched-chain VFA (isobutyrate + isovalerate + valerate); A:P ratio, acetate to propionate ratio. Kale varieties: K1, Caledonian; K2, Elba; K3, Sovereign; K4, Regal; K5, Coleor. Swede varieties: S1, Major Plus; S2, Aparima; S3, Highlander; S4, Dominion; S5, Invitation.
